# Development of a quantitative pharmacodynamic assay for apoptosis in fixed tumor tissue and its application in distinguishing cytotoxic drug—induced DNA double strand breaks from DNA double strand breaks associated with apoptosis

**DOI:** 10.18632/oncotarget.24936

**Published:** 2018-03-30

**Authors:** Angie B. Dull, Deborah Wilsker, Melinda Hollingshead, Christina Mazcko, Christina M. Annunziata, Amy K. LeBlanc, James H. Doroshow, Robert J. Kinders, Ralph E. Parchment

**Affiliations:** ^1^ Clinical Pharmacodynamic Biomarkers Program, Applied/Developmental Research Directorate, Leidos Biomedical Research, Frederick National Laboratory for Cancer Research, Frederick, Maryland, USA; ^2^ Biological Testing Branch, National Cancer Institute-Frederick, Frederick, Maryland, USA; ^3^ Comparative Oncology Program, National Cancer Institute, Bethesda, Maryland, USA; ^4^ Women's Malignancies Branch, National Cancer Institute, Bethesda, Maryland, USA; ^5^ Division of Cancer Treatment and Diagnosis and Center for Cancer Research, National Cancer Institute, Bethesda, Maryland, USA

**Keywords:** immunofluorescence, pharmacodynamic assay, DNA double strand breaks, multiplex, apoptosis

## Abstract

DNA double strand breaks (DSBs) induced by cancer therapeutic agents can lead to DNA damage repair or persistent DNA damage, which can induce apoptotic cell death; however, apoptosis also induces DSBs independent of genotoxic insult. γH2AX is an established biomarker for DSBs but cannot distinguish between these mechanisms. Activated cleaved caspase-3 (CC3) promotes apoptosis by enhancing nuclear condensation, DNA fragmentation, and plasma membrane blebbing. Here, we describe an immunofluorescence assay that distinguishes between apoptosis and drug-induced DSBs by measuring coexpression of γH2AX and membrane blebbing−associated CC3 to indicate apoptosis, and γH2AX in the absence of CC3 blebbing to indicate drug-induced DNA damage. These markers were examined in xenograft models following treatment with topotecan, cisplatin, or birinapant. A topotecan regimen conferring tumor regression induced tumor cell DSBs resulting from both apoptosis and direct DNA damage. In contrast, a cisplatin regimen yielding tumor growth delay, but not regression, resulted in tumor cell DSBs due solely to direct DNA damage. MDA-MB-231 xenografts exposed to birinapant, which promotes apoptosis but does not directly induce DSBs, exhibited dose-dependent increases in colocalized γH2AX/CC3 blebbing in tumor cells. Clinical feasibility was established using formalin-fixed, paraffin-embedded biopsies from a canine cancer clinical trial; γH2AX/CC3 colocalization analysis revealed apoptosis induction by two novel indenoisoquinoline topoisomerase I inhibitors, which was consistent with pathologist-assessed apoptosis and reduction of tumor volume. This assay is ready for use in clinical trials to elucidate the mechanism of action of investigational agents and combination regimens intended to inflict DNA damage, apoptotic cell death, or both.

## INTRODUCTION

The histone protein H2AX is phosphorylated on serine 139 to form γH2AX at sites of DNA double-strand breaks (DSBs), and this phosphorylation is required for the recruitment of DNA repair factors after DNA damage. Our lab and others have demonstrated the appearance of γH2AX after treatment with genotoxic agents by immunofluorescence assay using formalin-fixed, paraffin-embedded tumor tissue [1–3]. The γH2AX immunofluorescence assay (IFA) we developed for analysis of clinical specimens has been widely used across numerous NCI-sponsored clinical trials to reliably detect DSBs in tumor cells examined in clinical specimens. However, while the function of γH2AX was originally believed to be associated primarily with DNA repair, its role as a marker in DNA ladder formation during apoptosis has been well-established [4, 5]. Therefore, the observed γH2AX signal in clinical specimens could be indicative of apoptosis rather than DNA repair. We sought to resolve this ambiguity by developing an immunofluorescence microscopy assay to identify and enumerate γH2AX-positive cells undergoing apoptosis.

One hallmark of apoptosis is the cleavage-induced activation of caspase-3, an executioner caspase that promotes cell death by increasing the proteolysis of a variety of protein substrates. In the intrinsic apoptotic pathway, cytochrome *c*−mediated apoptosome formation induces procaspase-9 cleavage; activated caspase-9 then catalyzes the proteolytic processing of caspase-3, forming active cleaved caspase-3. Cleaved caspase-3 (CC3) is used as a marker of apoptosis in sandwich ELISA [6] and immunohistochemical analyses, but the detection of substantial levels of this activated caspase in many non-apoptotic cells [7–9] has confounded the use of CC3 alone as a specific, quantitative marker of apoptosis. Though the functional role of CC3 in non-apoptotic cells has not been fully explored, the presence of this protein may be due to its well-documented involvement in cell differentiation [10–16]. Thus, although CC3 is an integral executioner caspase essential for apoptosis, the alternate functions of this protein in non-apoptotic cells render it non-specific as a molecular biomarker of apoptosis.

However, in our efforts to resolve interpretation of the γH2AX immunofluorescence signal in tumor tissue, we observed formation of CC3 aggregates, or puncta, in cells undergoing apoptosis. Caspase-mediated cleavage of type I keratins during apoptosis is known to lead to formation of such CC3-containing puncta independent of γH2AX [4, 17, 18], and we found that these CC3 puncta were associated with the membrane blebbing that is characteristic of apoptotic cells (Supplementary Figure 1). By developing a microscopy method to enumerate cells containing both γH2AX and these blebbing-associated CC3 structures, we were able to quantitate apoptotic tumor cells harboring DNA fragmentation. The use of two independent biomarkers, including one that represents both a biochemical and morphological feature of apoptosis, results in an immunofluorescence assay with very high specificity and utility for assessing apoptosis in fixed tissue specimens on the preferred, microscopy platform for γH2AX evaluation. This method accomplishes two primary goals: first, to define the mechanism of action of novel cancer agents in clinical specimens in which a γH2AX response has been observed and, second, to report quantitative measurements of apoptosis in fixed tumor tissue.

Here, we present the development and validation of this high-specificity IFA for detection and enumeration of apoptotic cells in tumor tissue. We showed that using membrane blebbing−associated CC3 instead of total CC3 intensity to enumerate CC3-positive cells [“CC3(bleb)” IFA] substantially reduces background signal in tumor tissue from untreated canine patients. We applied this CC3(bleb) assay together with quantitation of nuclear γH2AX to distinguish apoptotic from non-apoptotic γH2AX-positive cell populations within tumors from xenograft models treated with the genotoxic agents cisplatin and topotecan in comparison to the pro-apoptotic agent birinapant. Finally, we demonstrated clinical readiness of this assay through its application to patients in a canine lymphoma clinical trial of investigational indenoisoquinoline topoisomerase I inhibitors. These results demonstrate the utility of our assay in dissecting the dynamics of DNA damage response and apoptosis in tumor tissue following treatment with different classes of anticancer therapeutics.

## RESULTS

### Identification and enumeration of cleaved caspase-3 blebbing−positive cells in tumor tissue

**Figure 1:**
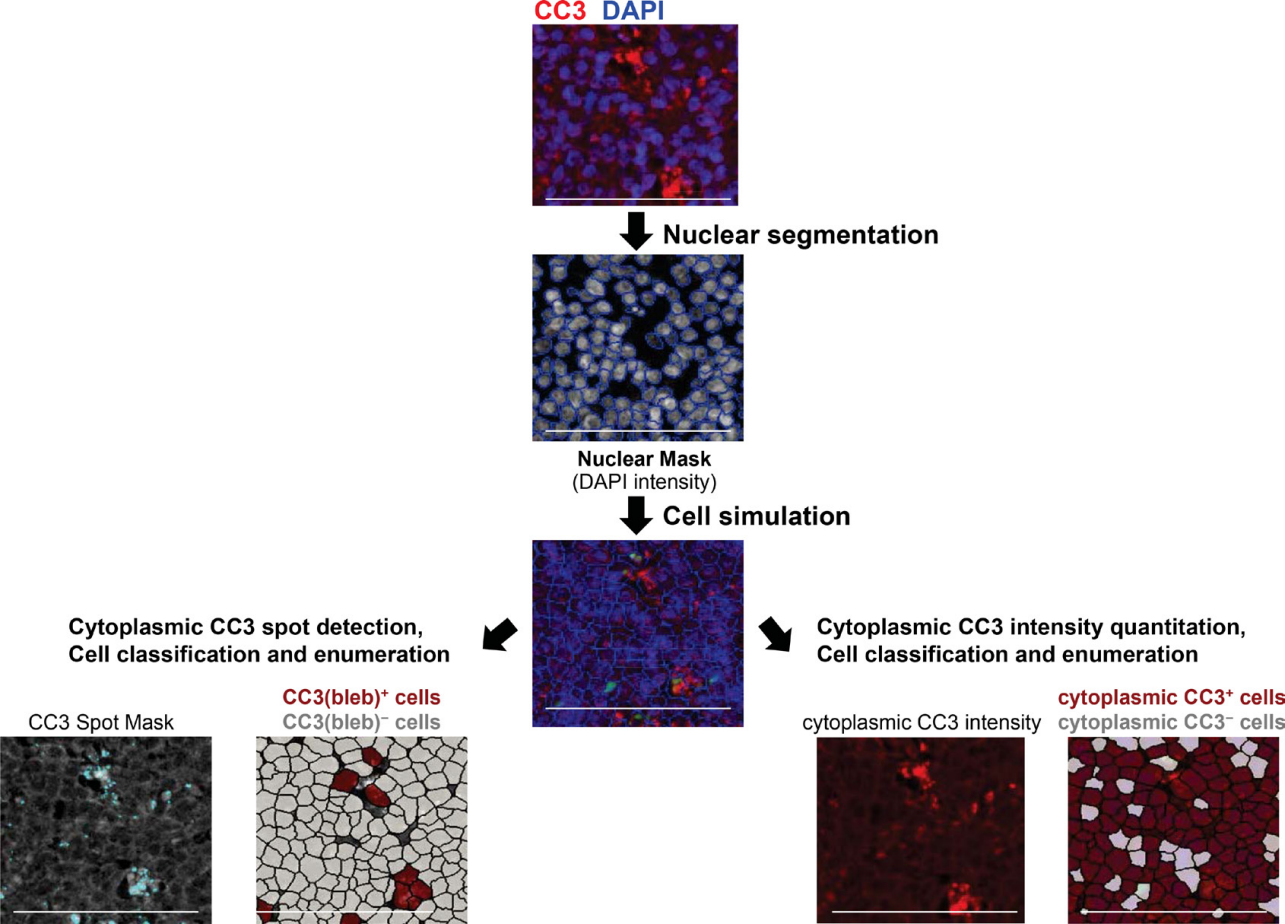
Workflow for enumeration of cleaved caspase-3 bleb−positive [CC3(bleb)^+^] cells in FFPE canine tumor tissue Images of tissue stained with DAPI and anti−cleaved caspase-3 antibody were first subjected to nuclear segmentation based on DAPI signal and nuclear size. Cytoplasmic areas were then simulated using a ring-based algorithm, defining the cytoplasm through a set dilation from the nuclear border (1–5 μm, established automatedly on a specimen-by-specimen basis). CC3 puncta were defined using a spot algorithm (left), with a spot size range of 0.2–5 μm^2^, and cells were classified as CC3(bleb)^+^ if they contained ≥2 CC3 puncta per cell. For comparison, cells were classified based on total cytoplasmic CC3 intensity (right). Scale bar represents 100 μm.

During apoptosis, caspase-mediated cleavage of type I keratins, such as K18, leads to the formation of large cytoplasmic inclusions, or puncta, containing keratins and CC3 [17, 18]. This process, together with other cytoskeletal changes, results in the characteristic membrane blebbing observed in apoptotic cells [19]. During our examination of tumor tissue from xenograft models and canine lymphoma biopsy specimens, we found that cells positive for CC3 puncta also exhibited membrane blebbing, with the latter visualized by immunofluorescence staining of the plasma membrane marker Na^+^/K^+^-ATPase (Supplementary Figure 1). We henceforth refer to cells containing CC3 puncta as CC3(bleb)^+^ cells, and we used a combination of ring-based nuclear border dilation and spot algorithms to identify and enumerate CC3(bleb)^+^ cells in formalin-fixed, paraffin-embedded (FFPE) tumor tissue (Figure 1).

**Figure 2:**
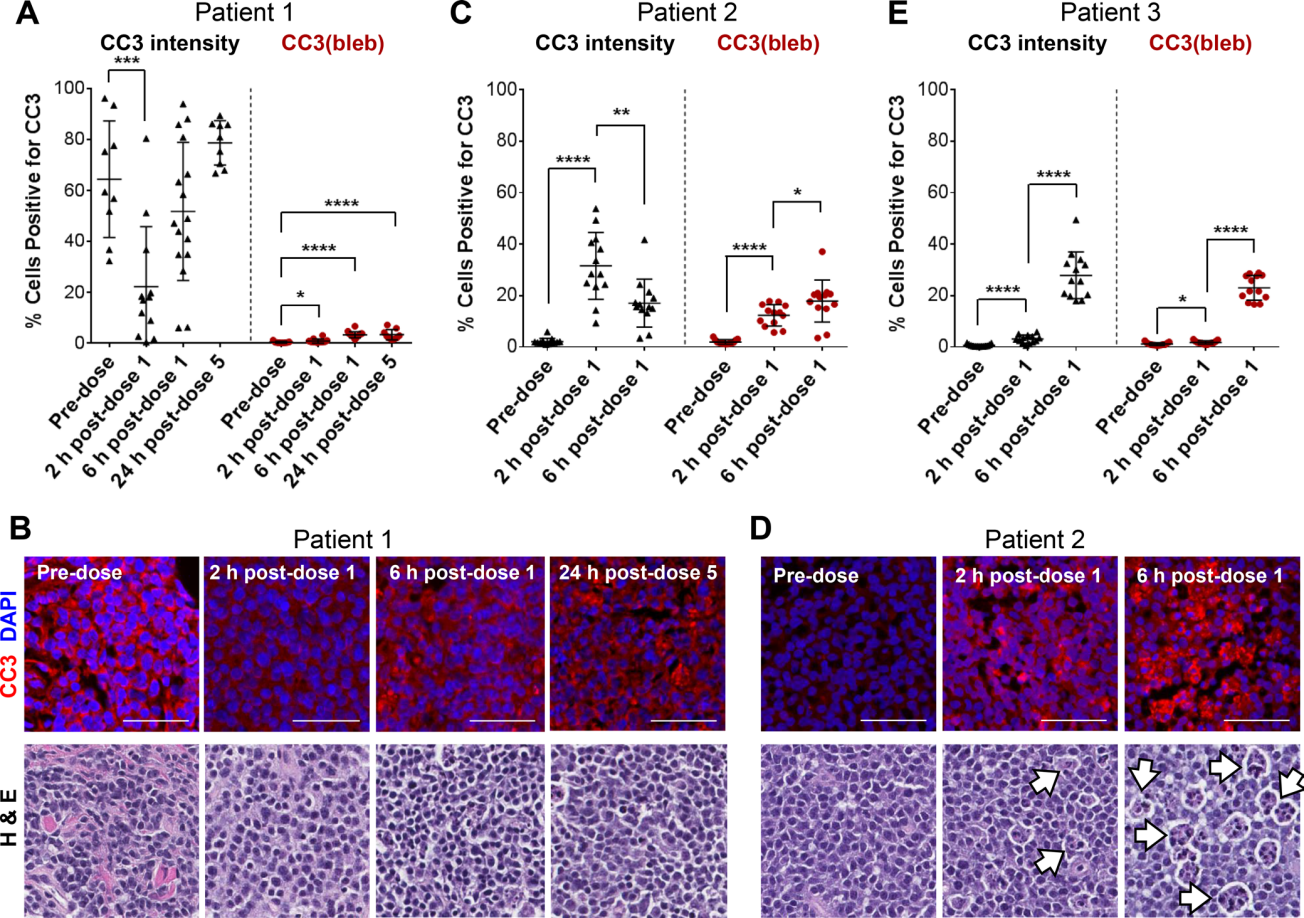
A CC3 blebbing mask improves the accuracy and precision of CC3 IFA−based apoptotic cell enumeration in FFPE tumor biopsy samples from canine lymphoma patients Needle biopsies were obtained 2 and 6 h post−dose 1 or 24 h post−dose 5 of QD × 5 treatment with the investigational indenoisoquinoline topoisomerase I inhibitors LMP744 (Patient 1), LMP400 (Patient 2), or LMP776 (Patient 3). (**A, C,** and **E**) Quantitation of cells positive for total cytoplasmic CC3 intensity (left, black) and CC3 blebbing (right, red) in tumor tissue collected at baseline and following treatment with investigational agents. Data are shown for 3 individual patients; each data point represents one image field, and lines indicate means ± standard deviations. Asterisks indicate statistically significant differences for comparisons with the pre-dose group (A) or for pre-dose vs. 2 h post-dose and 2 h vs. 6 h post-dose groups (C and E; ^*^P < 0.05, ^**^P < 0.01, ^***^P < 0.001, ^****^P < 0.0001). (**B** and **D**) Representative CC3/DAPI IFA and H & E images for patients 1 and 2. Scale bars represent 50 μm. White arrows indicate representative, pathologist-annotated “starry-sky” tumor-associated macrophages. IFA and H & E images from Patient 3 are presented in Supplementary Figure 2.

To examine whether our assay for CC3(bleb)^+^ cells provided a more accurate measure of apoptosis relative to quantitation of total cytoplasmic CC3, we assessed lymph node biopsy specimens from three canine patients with lymphoma who were enrolled in an ongoing clinical study of the investigational topoisomerase 1 inhibitors LMP400 (indotecan), LMP776 (indimitecan), and LMP744 (Figure 2). Measurements of diffuse cytoplasmic CC3 were highly variable and in some cases reported a signal in patients in which no apoptotic cells could be confirmed by a pathologist's assessment of hematoxylin and eosin (H & E)−stained slides (Figure 2A and 2B). In contrast, the CC3(bleb) assay accurately captured the pathologist-identified differences in apoptotic frequency between pre- and post-treatment samples.

Analysis of specimens from patient 1, treated with LMP744, illustrates these differences (Figure 2A). Cytoplasmic CC3 intensity quantitation indicates that 60% of cells in the pre-dose sample were positive for cytoplasmic CC3, that the percentage of cytoplasmic CC3^+^ cells decreased significantly (to approximately 20%; *P* < 0.001) 2 hours after administration of the first dose, and that the percentages of cytoplasmic CC3^+^ cells collected 6 hours post−dose 1 and 24 hours post−dose 5 were not significantly changed from before treatment (Figure 2A). In contrast, CC3(bleb) assay analysis yielded a mean of only 0.3% CC3(bleb)^+^ cells in the pre-dose sample, and very small but statistically significant increases in the percentage of CC3(bleb)^+^ cells at 2 and 6 hours post−dose 1 and 24 hours post−dose 5 (to 0.8%, 3.3%, and 3.3%, respectively; *P* < 0.05). These CC3(bleb) assay results reflect the absence of an appreciable number of apoptotic cells observed in the H & E images of these specimens (Figure 2B). Discrepancies between the cytoplasmic CC3 and CC3(bleb) assay results were also observed in specimens collected from patient 2, treated with LMP400 (Figure 2C); cytoplasmic CC3 measurements indicated that the percentage of cytoplasmic CC3^+^ cells significantly decreased from 2 hours to 6 hours post−dose 1 (31.6% to 17.1%, respectively; *P* < 0.01). In contrast, the CC3(bleb) assay results indicated a statistically significant *increase* in CC3(bleb)^+^ cells over this same time frame (from 12.3% to 17.9%; *P* < 0.05), consistent with the increase in apoptotic cells that can be observed in H & E images for the 2- and 6-hour post−dose 1 specimens (Figure 2D). This increase in apoptotic cells at 6 hours post−dose 1 is also consistent with enhanced numbers of “starry sky” tumor-associated macrophages (Figure 2D), which are known to associate with apoptotic cells within some lymphoma tumors [20].

For patient 3, treated with LMP776, the relative changes in apoptotic frequency were similar when quantitated by cytoplasmic CC3 intensity or CC3 blebbing (Figure 2E and Supplementary Figure 2), but in none of the cases examined did the cytoplasmic CC3 intensity measurements outperform the CC3(bleb) assay in terms of corresponding with the pathologist's assessment of apoptotic frequency. The CC3(bleb) assay also improved the precision of CC3 positivity measurements in that, for all three patients, variations in the CC3(bleb) signal (i.e., standard deviations provided in Figure 2) were smaller than those for total cytoplasmic CC3 intensity at all of the post-treatment time points examined. These data indicate that quantitation of cytoplasmic CC3 intensity is not a suitable approach for incorporation into an assay developed to measure apoptosis and that measurement of CC3 blebbing offers improved specificity for detection of apoptotic cells.

### Colocalization of γH2AX with CC3 blebbing distinguishes apoptosis-associated versus DNA damage-induced double-strand breaks

**Figure 3:**
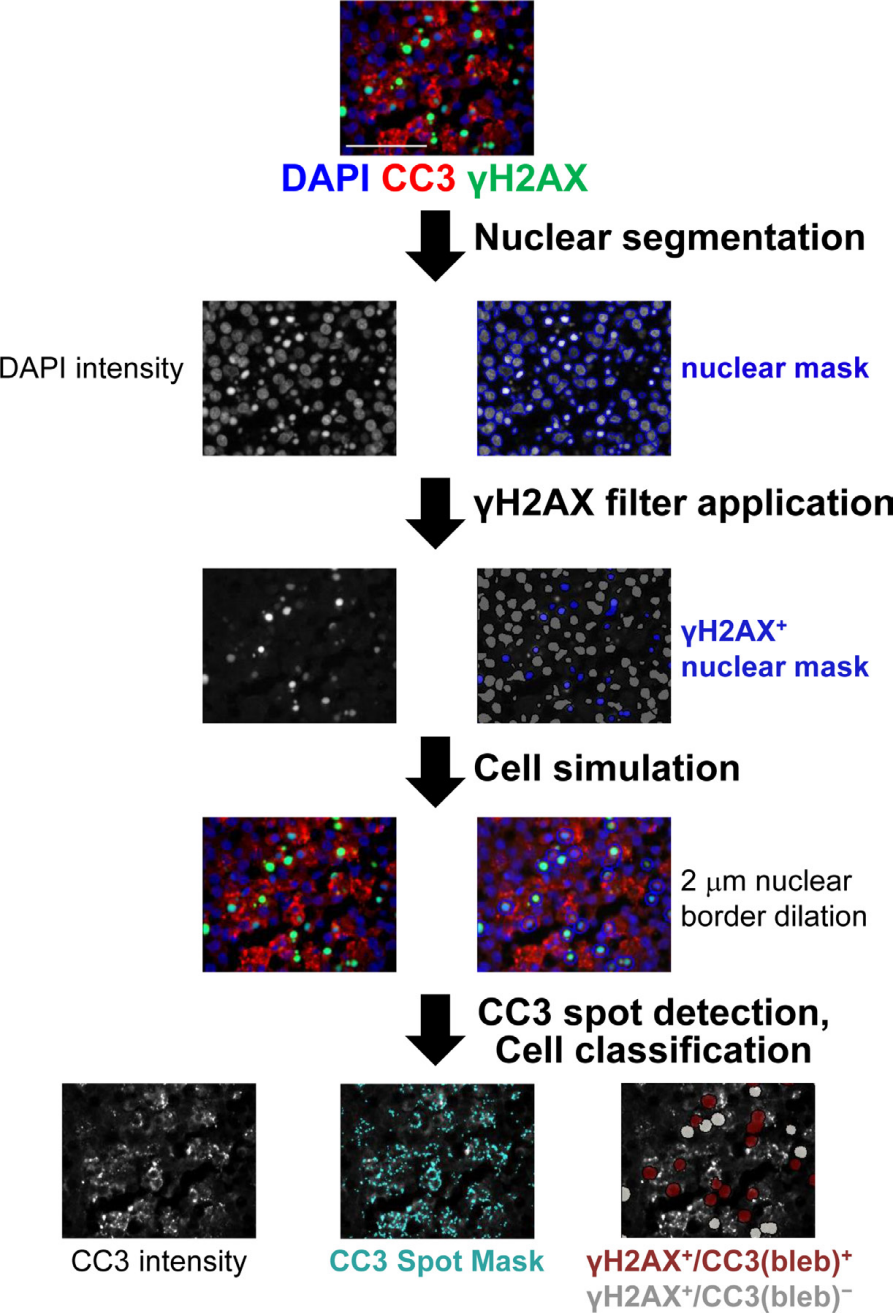
Workflow for quantitation of γH2AX^+^/CC3(bleb)^+^ cells in FFPE canine tumor tissue Tissue stained with DAPI and anti-CC3 and anti-γH2AX antibodies was subjected to nuclear segmentation based on the DAPI signal. γH2AX^+^ nuclei were defined based on nuclear γH2AX expression, and then cytoplasmic areas were simulated using a ring-based algorithm, with the cytoplasm defined through a set dilation from the nuclear border (1–5 μm, established in an automated fashion on a specimen-by-specimen basis). CC3 puncta in these γH2AX-positive cells were defined using a spot algorithm for CC3 intensity, with a spot size range of 0.2–5 μm^2^, and cells were classified as CC3(bleb)^+^ if they contained ≥2 CC3 puncta per cell. Scale bar represents 50 μm.

To evaluate whether the strong γH2AX signal measured in some of our clinical specimens reflected drug-induced DNA DSBs or apoptosis, we explored the utility of combining CC3 blebbing analysis with our previously validated IFA for γH2AX [1, 21]. Measuring coexpression of these markers within multiple, individual cells (Figure 3) allowed us to interpret whether γH2AX expression in a given cell is indicative of DNA damage due to genotoxic insult, or the induction of apoptosis and the associated genome-wide chromosomal DNA fragmentation [22]. Cellular colocalization of γH2AX and CC3(bleb) signals provides a highly specific marker of apoptosis, indicating apoptotic cells that have undergone DNA fragmentation.

**Figure 4:**
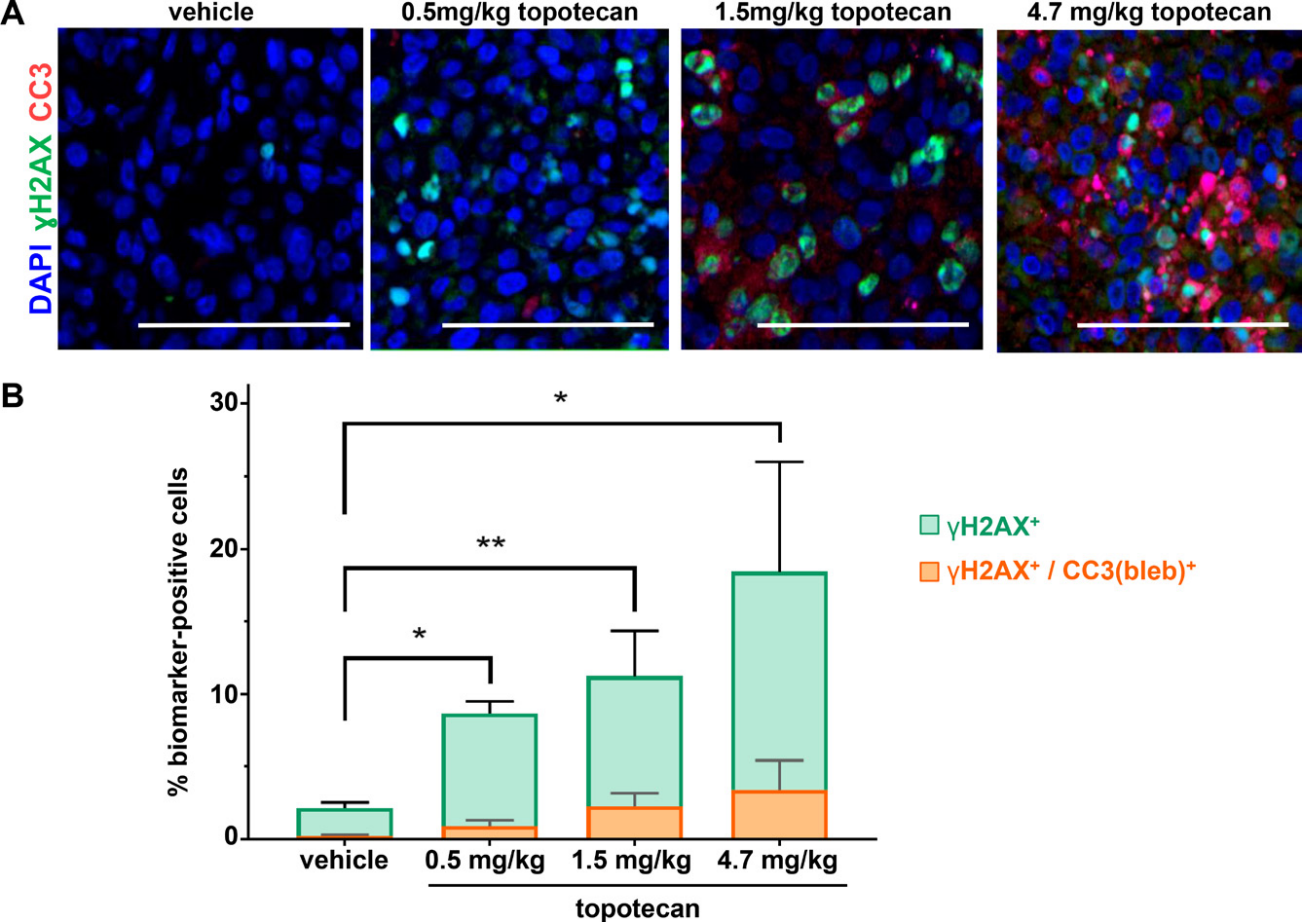
γH2AX/CC3(bleb) cellular colocalization assay results reveal dose-dependent induction of apoptosis at topotecan levels associated with tumor growth delay in the A375 melanoma xenograft model Mice were administered the indicated doses of topotecan, and tumors were harvested for analysis at 4 hours post dose (*n* = 4–6 animals per treatment group). (**A**) Representative IFA images from tumor tissue stained with anti-CC3 (red), anti-γH2AX (green), and DAPI (blue) are shown for each treatment group; scale bar represents 100 μm. (**B**) Mean total percentages of topotecan-treated A375 xenograft tumor cells positive for γH2AX (green) or γH2AX/CC3 blebbing cellular colocalization (orange) based on γH2AX/CC3(bleb) IFA quantitation of tumor sections from animals treated at the indicated doses (bars representing γH2AX and γH2AX/CC3 [bleb] are overlaid for comparison). Mean values from 4-6 animals (and 8 image fields per animal) are shown for each group; error bars indicate standard deviation. Asterisks indicate statistically significant differences (^*^P < 0.05, ^**^P < 0.01); levels of significance were identical for comparisons of both γH2AX and γH2AX/CC3(bleb).

We first applied the γH2AX/CC3(bleb) assay to examine the induction of DNA damage response and apoptosis pathways following treatment with the genotoxic DNA topoisomerase I inhibitor topotecan in the A375 melanoma xenograft model (Figure 4A). Administration of 1.5 or 4.7 mg/kg topotecan has been shown to induce robust tumor γH2AX expression and dose-dependent tumor regression [1]. Application of the γH2AX/CC3(bleb) assay to xenograft tumor samples collected 4 hours after topotecan treatment confirmed this dose-dependent increase in γH2AX (Figure 4B). At the two highest dose levels of topotecan, which were associated with antitumor efficacy, approximately 20% of the γH2AX^+^ cells were also positive for CC3 blebbing at 4 hours post administration, indicating that an appreciable portion of the total γH2AX signal is associated with apoptotic nuclei undergoing DNA fragmentation. At the lowest dose level and for the vehicle-treated group, the total γH2AX signals were lower, and CC3 blebbing was observed in only 10% of the γH2AX^+^ cells (Figure 4B). The significant increase in the percentage of γH2AX^+^/CC3(bleb)^+^ cells for the 1.5 and 4.7 mg/kg topotecan groups relative to vehicle is consistent with the observed efficacy differences for these groups and suggests that dose-dependent antitumor efficacy is associated with induction of both γH2AX and apoptosis, as would be expected for a DNA damaging agent. While the single time point examined may not represent peak γH2AX^+^/CC3(bleb)^+^ coexpression, this assay displays the sensitivity to detect even low levels of marker coexpression.

**Figure 5:**
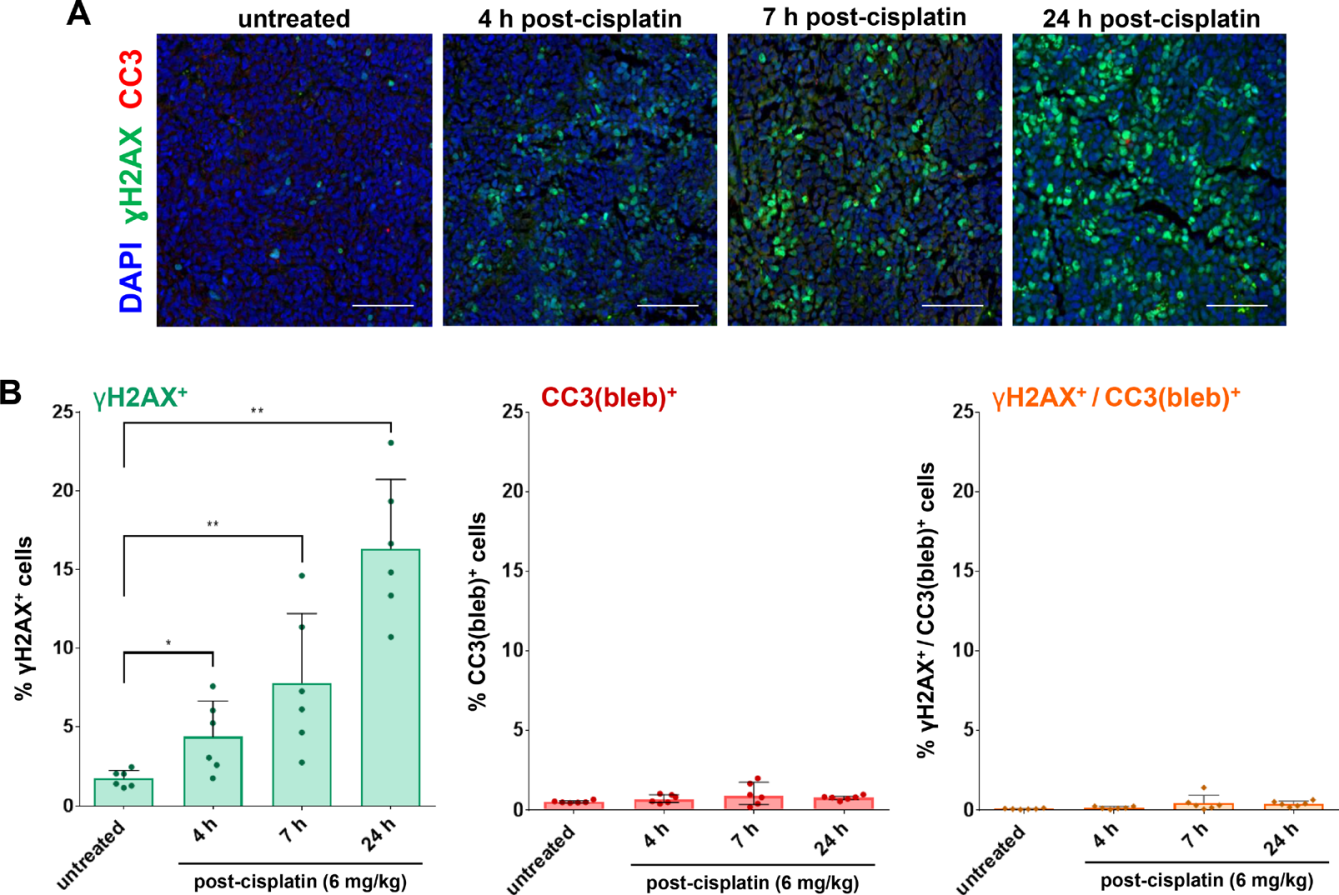
γH2AX/CC3(bleb) cellular colocalization assay results reveal induction of DNA damage response, but not apoptosis, following treatment with a single dose of a cisplatin regimen associated with slowed tumor growth in the A2780 ovarian carcinoma xenograft model Mice bearing A2780 xenografts were treated with a single dose of 6 mg/kg cisplatin, and tumors were harvested for γH2AX/CC3(bleb) IFA analysis at the indicated time points after cisplatin administration (*n* = 6 animals per time point). (**A**) Representative IFA images show cleaved caspase-3 (red), γH2AX (green), and DAPI (blue) staining in sections of FFPE tumor tissue; scale bars represent 100 μm. (**B**) Percentages of cisplatin-treated A2780 xenograft tumor cells positive for nuclear γH2AX (green, left), CC3 blebbing (red, center), and cellular colocalization of γH2AX and CC3 blebbing (orange, right), based on γH2AX/CC3(bleb) IFA quantitation of tumor sections. Each data point represents the average of 6 image fields from each animal in each treatment group (error bars represent standard deviation). Asterisks indicate statistically significant differences for comparisons with the untreated control group (^*^P < 0.05, ^**^P < 0.01).

We next examined γH2AX expression following treatment with the DNA crosslinking agent cisplatin in the A2780 ovarian carcinoma xenograft model over a 24-hour time course. A single 6 mg/kg dose of cisplatin was previously shown to slow, but not inhibit, tumor growth in this model. Our analysis of tumor quadrants harvested from A2780 models treated with this dose revealed significant increases in the percentage of γH2AX^+^ cells at 4, 7, and 24 h post-cisplatin administration, but no significant changes in the percentage of CC3(bleb)^+^ or γH2AX^+^/CC3(bleb)^+^ cells at any time point up to 24 hours (Figure 5). Similar to the pattern of γH2AX expression in the topotecan-treated xenografts, the γH2AX signal observed following cisplatin treatment was pan-nuclear rather than focal, indicating that the spatial pattern of γH2AX expression does not necessarily offer any additional information to distinguish between persistent DNA damage and apoptosis induced by genotoxic agents. The induction of only γH2AX following a single 6 mg/kg dose of cisplatin is consistent with the modest tumor growth delay observed following this treatment [23] and suggests that this dose causes steady accumulation of DNA DSBs that do not trigger apoptosis even at 24 hours.

### Colocalization of γH2AX and CC3 blebbing is a biomarker for the antitumor activity of the pro-apoptotic agent birinapant

**Figure 6:**
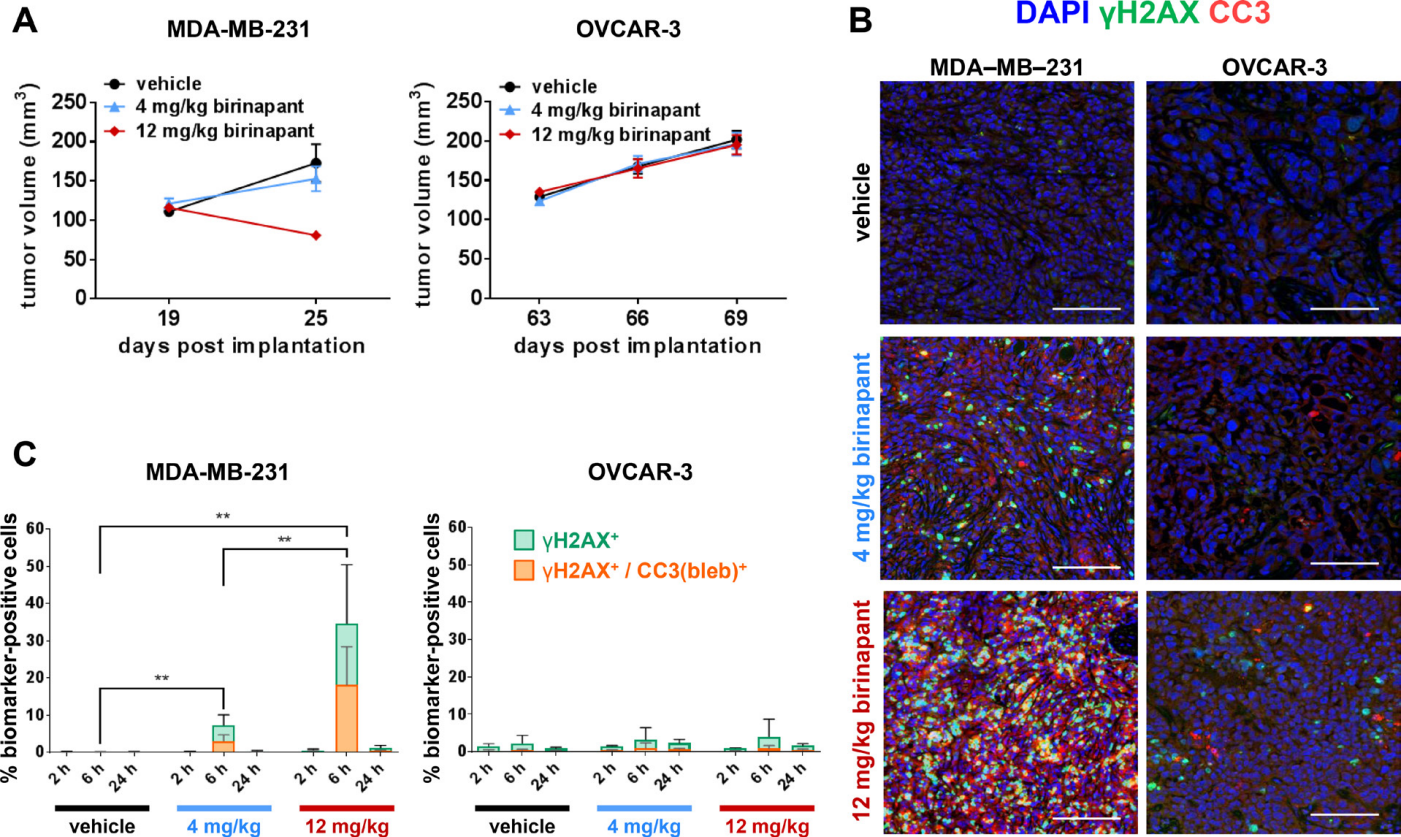
γH2AX/CC3(bleb) colocalization is a high-specificity biomarker for *in vivo* antitumor activity of the apoptosis-inducing agent birinapant (**A**) Median tumor volumes (± standard error of the median) on the indicated days are shown for MDA-MB-231 breast adenocarcinoma (left) and OVCAR-3 ovarian adenocarcinoma (right) xenograft models treated on post-implantation days 19, 22, and 25 (MDA-MB-231) or 63, 66, and 69 (OVCAR-3) with vehicle (black circles), 4 mg/kg birinapant (blue triangles), or 12 mg/kg birinapant (red diamonds); these data were published previously [6] (*n* = 17–18 animals per treatment group for each model). (**B**) and (**C**) MDA-MB-231 or OVCAR-3 xenograft models were treated with a single dose of vehicle or 4 or 12 mg/kg of birinapant, and tumors were harvested for γH2AX/CC3(bleb) IFA analysis at 2, 6, or 24 hours after birinapant administration (*n* = 6 animals per treatment group per time point). (**B**) Representative IFA images are shown for sections of FFPE tumor tissue from MDA-MB-231 (left) or OVCAR-3 (right) xenograft tumors harvested at 6 hours post-dose and stained for cleaved caspase-3 (red), γH2AX (green), or with DAPI (blue); scale bars represent 100 μm. (**C**) Mean percentages of MDA-MB-231 (left) or OVCAR-3 (right) xenograft tumor cells positive for nuclear γH2AX (green) or cellular colocalization γH2AX and CC3 blebbing (orange) for each treatment group and time point based on quantitation of IFA images (bars representing γH2AX and γH2AX/CC3[bleb] are overlaid for comparison). Bars indicate mean values from 6 animals (and 10 image fields per animal) in each treatment group; error bars represent standard deviation. Asterisks indicate statistically significant differences (^**^P < 0.01); levels of significance were identical for comparisons of both γH2AX and γH2AX/CC3(bleb).

Birinapant is a mimetic of the proapoptotic protein Smac (second mitochondrial activator of caspases), which deactivates inhibitors of apoptosis (IAP) such as survivin, XIAP (X-chromosome-linked IAP) and cIAP1/2 (cellular IAP1/2) [6, 24–27]. Results from previous experiments [7] showed that treatment with 4 or 12 mg/kg birinapant (Q3D × 3) slowed or inhibited the growth of MDA-MB-231 breast carcinoma xenograft model tumors but had no effect on the growth of OVCAR-3 ovarian carcinoma xenograft model tumors (Figure 6A). Reflecting this differential efficacy, analysis of tumor quadrants harvested after administration of a single 4 or 12 mg/kg birinapant dose revealed a substantial increase in the percentage of γH2AX^+^/CC3(bleb)^+^ cells 6 hours after treatment in MDA-MB-231, but not OVCAR-3, xenografts; signal in the MDA-MB-231 tumors returned to baseline by 24 hours at both dose levels (Figure 6B and 6C). Together, these results serve as proof-of-concept, demonstrating that the degree of γH2AX^+^/CC3(bleb)^+^ cell induction observed 6 hours after treatment with birinapant follows the degree of antitumor activity of this agent in the MDA-MB-231 and OVCAR-3 xenograft models.

### Induction of apoptosis by two novel indenoisoquinolines in canine clinical specimens

**Figure 7:**
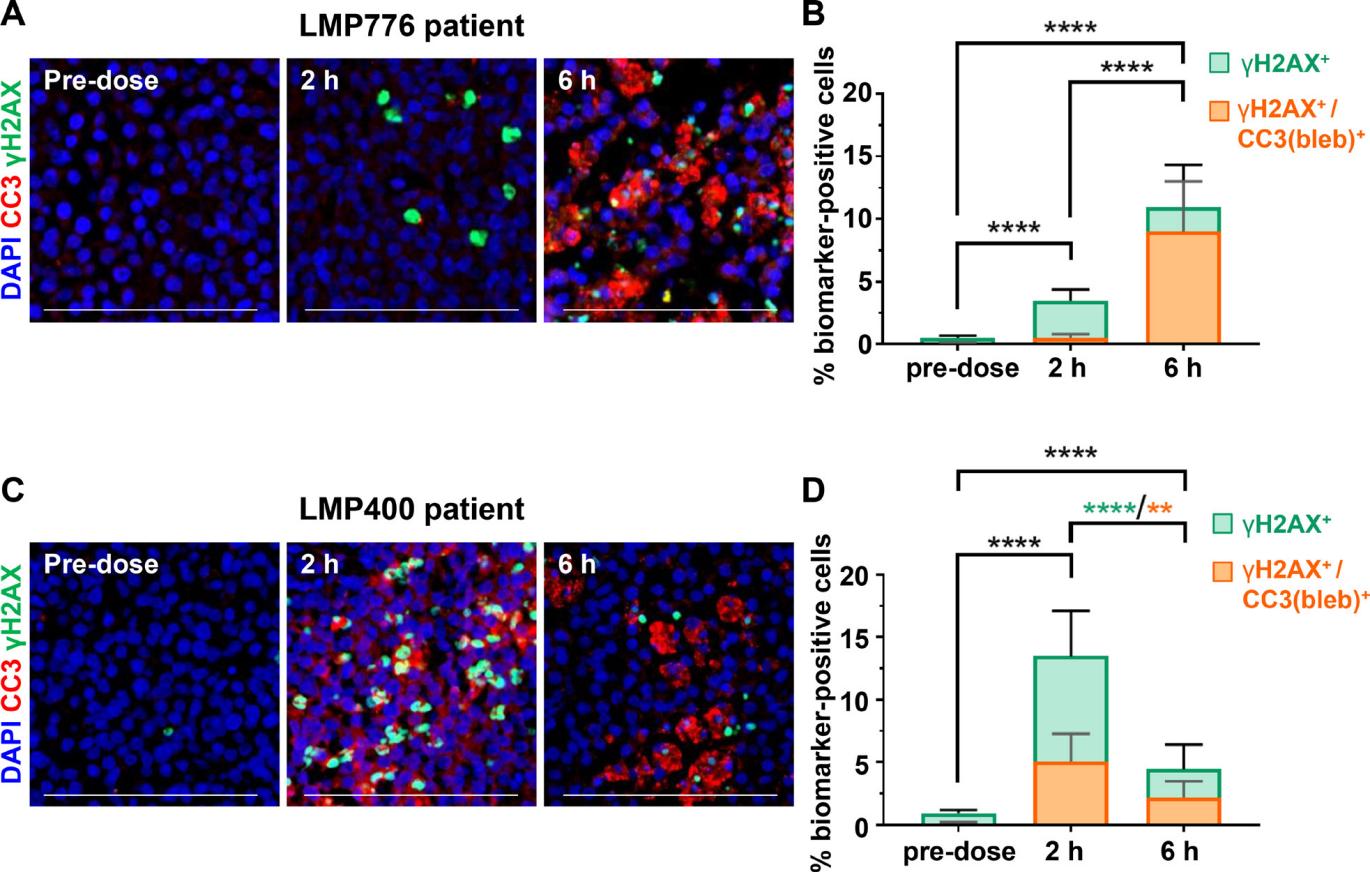
Analysis of the γH2AX/CC3(bleb) biomarker response to indenoisoquinoline treatment in core needle biopsies from canine lymphoma patients Eighteen-gauge tumor needle biopsies were harvested from involved lymph nodes of 2 lymphoma patients. (**A**) and (**C**) Representative IFA images of sections from FFPE needle biopsies obtained at the indicated times on day 1 of QD × 5 treatment with 17.5 mg/m^2^ LMP776 (A) or 65 mg/m^2^ LMP400 (C); staining shows cleaved caspase-3 (red), γH2AX (green), and DAPI (blue), and scale bars represent 100 μm. (**B**) and (**D**) Quantitation of the γH2AX (green) and γH2AX/CC3(bleb) cellular colocalization (orange) signals corresponding to the images shown in (A) and (C), respectively. Bars indicate mean values from 13 image fields, and bars representing γH2AX and γH2AX/CC3 (bleb) are overlaid for comparison; error bars represent standard deviation. Asterisks indicate statistically significant differences (^**^P < 0.01, ^****^P < 0.0001) for comparisons of both γH2AX and γH2AX/CC3(bleb) (black) or γH2AX only (green) or γH2AX/CC3(bleb) (orange). The patient from which the data in (A) and (B) were obtained is Patient 3 from Figure 2E; the patient featured in (C) and (D) is Patient 2 from Figure 2C.

Clinical feasibility of the γH2AX/CC3(bleb) assay was established by analyzing specimens from a clinical trial of the investigational indenoisoquinoline topoisomerase 1 inhibitors indotecan (LMP400) and indimitecan (LMP776) in canine lymphoma patients. Each of the specimens had been identified as positive for nuclear γH2AX during an initial analysis—a step that lends itself well to the workflow associated with large trials testing genotoxic agents as it allows the duplex staining and advanced image analysis to be applied only to the subset of patient samples for which additional mechanistic data can be obtained. Two patients with tumors exhibiting clear evidence of apoptosis, as detected by the γH2AX/CC3(bleb) assay, are shown in Figure 7; these patient tumors exhibited maximum apoptotic responses of 5–9% of cells positive for γH2AX/CC3(bleb) colocalization, reflecting the corresponding clinical responses of > 60% reductions in tumor volume. In contrast, seven canine patients that had < 22% reductions in tumor volume showed < 1.5% γH2AX^+^/CC3(bleb)^+^ cells at the 2- and 6-hour time points (data not shown). Furthermore, at the time points of maximum total γH2AX response for the patient tumors shown in Figure 7, a substantial portion of the γH2AX^+^ cells were apoptotic (82% and 38% for the patient tumors shown in Figure 7B and 7D, respectively), highlighting the role of this assay in deconvoluting the ambiguous γH2AX signal in clinical specimens. In all of our analyses of canine patient specimens, we performed high-content imaging and analysis for a minimum of 15,000 individual cells per biopsy specimen. Collectively, the data presented for the specimens in Figure 2 and Figure 7 demonstrate that this quantity of cells is sufficient to detect significant increases in CC3 blebbing and γH2AX/CC3(bleb) colocalization.

These canine patient samples also illustrate the utility of this assay in enabling temporal dissection of the DNA damage repair and apoptotic responses. For the patient treated with LMP776 (Figure 7A and 7B), the presence of γH2AX^+^ cells but very few γH2AX^+^/CC3(bleb)^+^ cells at the 2-hour time point indicates a DNA damage repair response without appreciable apoptosis; in contrast, the significant increase in γH2AX positivity at the 6-hour time is due primarily to apoptotic cells (with 82% of γH2AX^+^ cells also positive for CC3 blebbing). The temporal pattern of DNA damage repair response relative to apoptosis differed for the patient treated with LMP400 (Figure 7C and 7D). In this case, the peak γH2AX^+^-only and peak γH2AX^+^/CC3(bleb)^+^ responses both occurred at the 2-hour time point and then decreased at 6 hours, suggesting that the maximum apoptotic response may occur earlier for LMP776 compared to LMP400; however, whether these temporal differences in biomarker response are due to interpatient heterogeneity or differing drug pharmacokinetics and/or mechanisms of action remains to be determined upon completion of this clinical trial. Together, these data collected from core needle biopsies of naturally occurring lymphomas provide a fitness-for-purpose demonstration that indicates the assay is suitable for further use in pharmacodynamically driven clinical trials of DNA damaging or pro-apoptotic agents.

## DISCUSSION

The ability to definitively interpret a positive γH2AX signal in tumor tissue improves our mechanistic understanding of investigational anticancer agents undergoing preclinical and clinical evaluation. Though there are numerous examples of γH2AX positivity in preclinical and clinical specimens, a positive γH2AX signal has been associated both with tumor volume reduction and tumor progression. Evaluating such specimens with the dual γH2AX/CC3(bleb) assay presented here allows us to interpret this ambiguous γH2AX positivity. Because scoring of γH2AX-expressing cells on a microscopy platform is the preferred method for DSB detection, we developed a microscopy assay to enumerate cells containing both γH2AX and blebbing-associated CC3 morphological structures in clinical specimens. By assessing the population of cells coexpressing both of these markers following efficacious therapies, we were able to evaluate the minimum proportion of apoptotic cells that is associated with efficacy at specific time points in these models. The γH2AX^+^/CC3(bleb)^+^ population in any given tumor sample is the result of a variety of factors, including the timing of induction for the two independent markers and the tumor cell kinetics of each drug; however, the γH2AX/CC3(bleb) assay is highly sensitive and allows for detection of even relatively low levels of apoptosis.

We report modulation of γH2AX/CC3(bleb) cellular colocalization and apoptosis in a manner associated with tumor volume reduction in all of the drug treatment settings examined. Treating the A375 xenograft model with topotecan increased apoptosis in a dose-dependent manner that was associated with tumor volume reduction (Figure 4 and [1]). On the other hand, treating A2780 xenograft−bearing mice with cisplatin induced significant DNA damage, as indicated by γH2AX-positive cells, without induction of apoptosis or tumor shrinkage [23]. Our xenograft studies with the cIAP inhibitor birinapant demonstrated extensive dose-dependent induction of apoptosis and antitumor efficacy in the sensitive MDA-MB-231 xenograft tumor model but not in the drug-resistant OVCAR-3 xenograft model. These preclinical birinapant data are consistent with those from a recent phase II clinical trial of birinapant in women with relapsed platinum-resistant or refractory epithelial ovarian cancer, in which qualitative assessment of two sets of paired biopsies revealed very few cells displaying colocalization of γH2AX and cytoplasmic CC3, consistent with the lack of efficacy of single-agent birinapant in this patient population [28]. Finally, clinical application of the γH2AX/CC3(bleb) assay to needle biopsy specimens collected from canine lymphoma patients treated with indenoisoquinolines revealed significant γH2AX/CC3(bleb) colocalization in post-treatment tumor specimens from two patients experiencing tumor shrinkage—and no such γH2AX/CC3(bleb) colocalization in tumors that were not responsive to these agents—further evidence that this assay is a reliable readout of cell death associated with tumor reduction.

We have developed a workflow to incorporate the γH2AX/CC3(bleb) assay into pharmacodynamic analyses of specimens from early-phase clinical trials involving DNA damage−inducing agents. The primary pharmacodynamic endpoint for genotoxic agents is induction of markers of DNA damage repair. Thus, rigorous analysis of multiple DNA damage repair markers (e.g., as detected by our γH2AX, phospho-Nbs1, and Rad51 multiplex immunofluorescence assays) is an essential first step when analyzing specimens from studies of DNA damage−inducing agents. In an effort to preserve valuable patient tissue specimens for the most informative biomarker analyses, our current workflow for such trials prioritizes the primary pharmacodynamic endpoint by first performing multiplex assays of DNA damage repair markers—a process that often requires extensive analysis. Only if γH2AX induction is observed in a specimen is it assessed for apoptosis as a tertiary biomarker in order to decipher the meaning of the γH2AX signal; specimens for which no γH2AX is detected may be used instead for other exploratory biomarker analyses. However, for trials in which apoptosis is an appropriate primary pharmacodynamic endpoint (e.g., studies of birinapant), it may be preferable to begin with the γH2AX/CC3(bleb) assay as a first step of specimen analysis. In such cases, extensive preclinical testing would be required to demonstrate that the agent of interest directly induces apoptosis rather than the apoptosis arising as a secondary event of widespread DNA damage.

While other microscopy-based methods of detecting apoptosis exist, such as TUNEL (terminal deoxynucleotidyl transferase dUTP nick-end labeling), these have limited quantitative capabilities due to insufficient signal-to-noise ratios—an issue that we have resolved via quantitation of CC3 blebbing, a marker of both the biochemical and morphological changes associated with apoptosis. Other quantitative, high-specificity assays for biomarkers of apoptosis exist in the multiplex immunoassay arena [6], but these are designed for Luminex-based measurements of tumor lysates rather than immunohistochemical or IFA analysis of intact tumor tissue. Thus, our method for identifying and enumerating gH2AX/CC3(bleb)−containing cells represents an important advance in the quantitative, microscopy-based analysis of apoptosis in tumor tissue. Our methodology can be applied as a highly specific apoptosis assay designed for IFA with fixed core needle biopsy specimens, and our results reveal important insights into mechanisms of action across multiple drug classes. This assay may have broad clinical and preclinical applicability to studies of investigational oncology agents and cytotoxic-based combination regimens.

## MATERIALS AND METHODS

### BOND-MAX Autostainer staining protocol

BOND-MAX staining methods have been previously described (21). The following antibody working solutions were prepared and loaded into the BOND-MAX Processing Module: a) 10 μg/mL rabbit monoclonal anti−cleaved caspase-3 antibody (R&D systems, MAB835, clone 269518, Milan, Italy) prepared in Bond Primary Antibody Diluent (Leica Biosystems, Buffalo Grove, IL), and goat-anti-rabbit Alexa Fluor 546 antibody (Invitrogen, Life Technologies, Grand Island, NY) prepared 1:100 in 1× Bond Wash Solution; b) 5 μg/mL monoclonal anti−phospho (Ser 139) γH2AX-FITC conjugate (Millipore, 16-202a, clone JBW301, Billerica, MA) antibody prepared in Bond Primary Antibody Diluent (Leica Biosystems, Buffalo Grove, IL); and c) 4′,6-diamidino-2-phenylindole (DAPI) dihydrochloride, FluoroPure™ grade (Invitrogen, Life Technologies, Grand Island, NY) prepared at 0.25 μg/mL in Bond Primary Antibody Diluent.

### Image capture and quantitation

Slides of xenograft and biopsy sections (5 μm) stained with the cleaved caspase-3/γH2AX/DAPI multiplex mixture were scanned using the Aperio ScanScope FL image capture system (Leica Biosystems); at least six fields from each specimen slide were analyzed. Images were acquired using 20X magnification with a Leica Plan Apo 20X/0.7NA objective, 0.4622 μm^2^/pixel resolution, and images had a 16-bit depth. The CC3 exposure range was 0.8–3 s, γH2AX exposure was 0.8–2 s, and DAPI exposure was 20–30 ms. Images were extracted with a fixed size (1,000 × 1,000 pixel) from full tissue scans using Aperio ImageScope software. Images selected for extraction were derived from tumor tissue determined to be of sufficient quality as assessed via evaluation of an adjacent H & E–stained section of each specimen by a board-certified pathologist [29]. Definiens Tissue Studio software (Definiens AG, Munich, Germany) was used for quantitative analysis of all xenograft and clinical biopsy material. Statistical significance was calculated by nonparametric Mann-Whitney tests. Proof of clinical readiness of the staining, image capture, and analysis was established with representative 18-gauge core needle biopsy specimens obtained from canine lymphoma patients.

### Cleaved caspase-3 intensity−based quantitation

Nuclei were segmented based on DAPI intensity signal; nuclear size among the different studies ranged from 50–200 μm^2^. Nuclei were filtered by area to remove regions of interest (ROIs) that were not properly segmented into single cell ROIs. The cytoplasmic mask was generated using a ring-based algorithm created using a set (1–5 μm) dilation from the nuclear border. CC3 intensity within the cytoplasmic mask was quantitated by setting an intensity threshold and using a classification based on this threshold intensity to define a positive cell. The output for this measurement is percentage of cells positive for cytoplasmic CC3.

### Cleaved caspase-3 blebbing quantitation

Nuclei were segmented and cytoplasmic masks generated as described above. CC3 puncta—a proxy for membrane blebbing (see Supplementary Figure 1)—were defined using a spot threshold algorithm for intensity and a spot size of 0.9 μm^2^; spots with an area less than 0.2 μm^2^ and greater than 5 μm^2^ were filtered out. Cells with 2 or more puncta were classified as positive. The output is percentage of cells positive for CC3 blebbing. A workflow for enumeration of CC3(bleb)^+^ cells is provided in Figure 1.

### Quantitation of γH2AX and cleaved caspase-3 blebbing cellular colocalization

Nuclei segmented as described above were filtered based on nuclear area and γH2AX intensity to remove ROIs that were not properly segmented into single cell ROIs and to select cells positive for γH2AX nuclear area signal using an automated custom intensity threshold [3]. Cytoplasmic masks were generated as described above. A spot algorithm was used to measure CC3 blebbing, as defined above, with cells containing 2 or more CC3 puncta per cell classified as positive. The output is percentage of cells positive for cellular colocalization of γH2AX and CC3 blebbing, where cells masked with red are defined as a positive and cells masked with white are negative. A workflow for enumeration of γH2AX^+^/CC3(bleb)^+^ cells is provided in Figure 3.

### Drug-treated xenograft models

Female athymic nu/nu mice were implanted with MDA-MB-231, A375, or A2780 human tumor cells as previously described [1, 6]. OVCAR-3 tumor cells were implanted into female NOD SCID gamma (NSG) mice as previously described [6]. A375 xenograft quadrants were collected from mice 4 hours after intraperitoneal administration of vehicle (sterile water) or 0.5, 1.5, or 4.7 mg/kg topotecan (NSC 609699). The maximum tolerated dose of topotecan administered to mice once a day for 5 consecutive days (QD × 5) is 4.7 mg/kg (14.1 mg/m^2^). A2780 xenografts were collected from mice following intraperitoneal administration of vehicle (saline) or 6 mg/kg cisplatin (NSC 119875) at 4, 7, or 24 hours after one dose. Dosing and sample collection for the birinapant studies are as described in [6]. All samples were fixed in neutral-buffered formalin and paraffin-embedded as previously described [1]. For the γH2AX/CC3(bleb) analyses of xenograft specimens, we analyzed ≥ 8,000 individual cells per specimen, which was sufficient to detect significant increases in CC3 blebbing and γH2AX/CC3(bleb) colocalization. Frederick National Laboratory is accredited by AAALAC International and follows the Public Health Service Policy for the Care and Use of Laboratory Animals. Animal care was provided in accordance with the procedures outlined in the “Guide for Care and Use of Laboratory Animals” (National Research Council; 2011; National Academies Press; Washington, D.C.). All studies were conducted under an approved Animal Care and Use Committee protocol. Mice were housed in sterile, filter-capped, polycarbonate cages (Allentown Caging) in a barrier facility on a 12-hour light/dark cycle, and provided with sterilized food and water, *ad libitum*. Prior to drug treatment, the animals were randomized into groups using a commercial software program (Study Director, Studylog Systems, Inc.).

### Canine clinical trial biopsy specimens

Patient specimens consisting of FFPE 18-gauge core needle biopsies from a canine clinical trial that accrued outbred dogs with naturally-occurring lymphomas were used to establish clinical feasibility. Collection and handling conditions for canine lymphoma core needle biopsies were based on specimen preservation and processing SOPs used for analysis in core needle biopsies of human tumors (https://dctd.cancer.gov/ResearchResources/ResearchResources-biomarkers.htm). Dogs were treated with one of three investigational indenoisoquinoline topoisomerase I inhibitors: LMP400 (indotecan), LMP776 (indimitecan), or LMP744 [30]. Core biopsies were collected from involved lymph nodes prior to treatment and at 2 and 6 hours following administration of the first dose of a QD × 5 regimen. An additional lymph node sample was collected 24 hours after completion of the 5-day regimen. After collection, all specimens were immediately flash-frozen and then fixed in 10% neutral buffered formalin (NBF) for 24 hours before paraffin-embedding (21). For the γH2AX/CC3(bleb) analyses of canine patient biopsy specimens, we analyzed ≥ 15,000 individual cells per specimen, which was sufficient to detect significant increases in CC3 blebbing and γH2AX/CC3(bleb) colocalization.

## SUPPLEMENTARY MATERIALS FIGURES



## Abbreviations

CC3: cleaved caspase-3
CC3(bleb): membrane blebbing−associated cleaved caspase-3
cIAP1/2: cellular IAP1/2
DAPI: 4′,6-diamidino-2-phenylindole
DSB: double strand break
FFPE: formalin-fixed, paraffin-embedded
γH2AX: S139-phosphorylated histone H2AX
H & E: hematoxylin and eosin
IAP: inhibitor of apoptosis
IFA: immunofluorescence assay
ROI: regions of interest
Smac: second mitochondrial activator of caspases
TUNEL: terminal deoxynucleotidyl transferase dUTP nick-end labeling
XIAP: X-chromosome-linked IAP
