# Supplementary material for: Development of a quantitative pharmacodynamic assay for apoptosis in fixed tumor tissue and its application in distinguishing cytotoxic drug—induced DNA double strand breaks from DNA double strand breaks associated with apoptosis
Source: Oncotarget. 2018 Mar 30;9(24):17104–16. doi: 10.18632/oncotarget.24936 (PMC5908309; doi:10.18632/oncotarget.24936)
Supplement: Supplementary file 1 [file oncotarget-09-17104-s001.pdf]

Development of a quantitative pharmacodynamic assay for apoptosis in fixed tumor tissue and its application in distinguishing cytotoxic drug−induced DNA double strand breaks from DNA double strand breaks associated with apoptosis

SUPPLEMENTARY MATERIALS

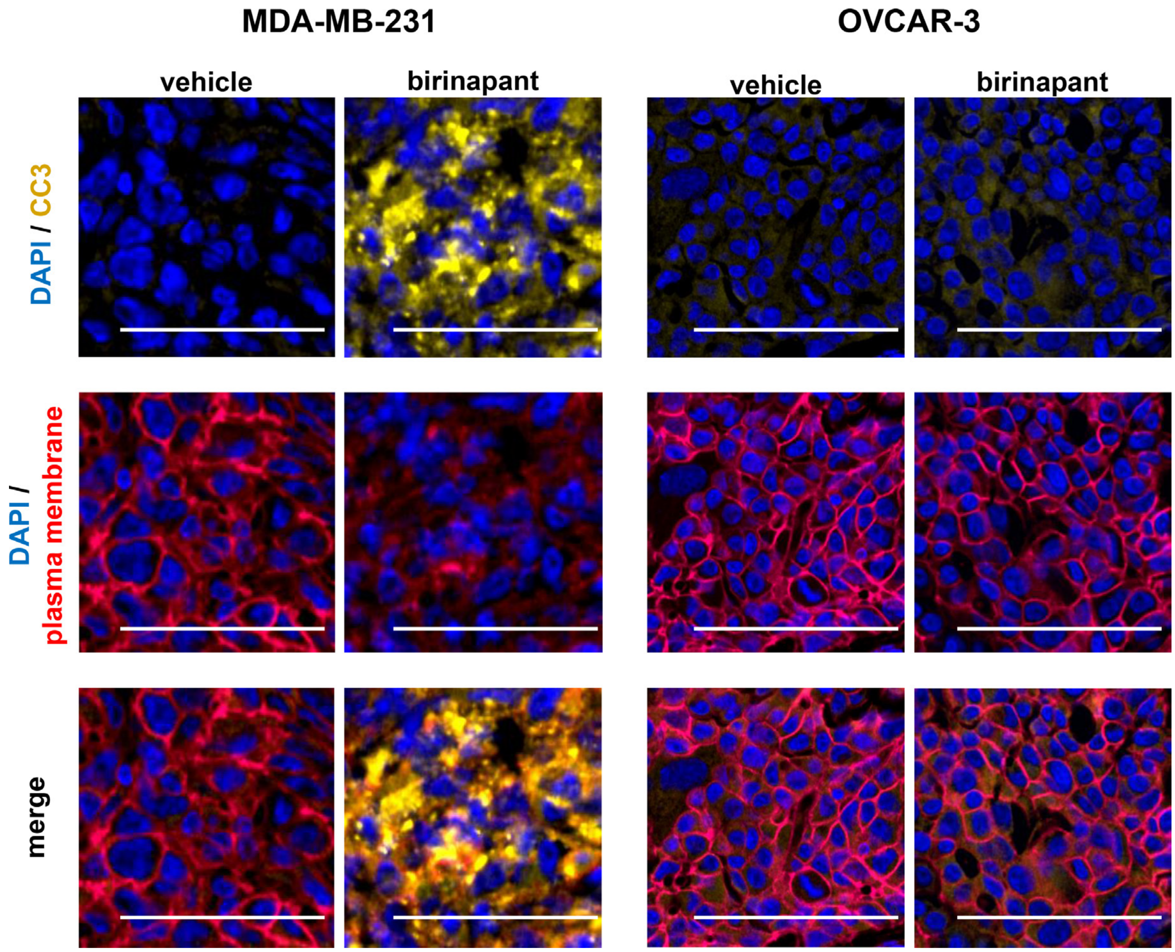

Supplementary Figure 1: CC3 puncta are associated with plasma membrane blebbing in tumor tissue from drug-treated xenograft models. Images of FFPE tumor tissue stained for CC3 (yellow), DAPI (blue), and the plasma membrane marker Na^+/K^+-ATPase (red) are shown for MDA-MB-231 or OVCAR-3 human tumor xenograft models 6 hours following treatment with vehicle (left) or 12 mg/kg of the apoptosis-inducing agent birinapant (right). Scale bars represent 50 µm. Colocalization of CC3 puncta with regions of plasma membrane blebbing in the responsive MDA-MB-231 model but not the resistant OVCAR-3 model indicates that CC3 puncta positivity is associated with membrane blebbing, a hallmark of apoptosis.

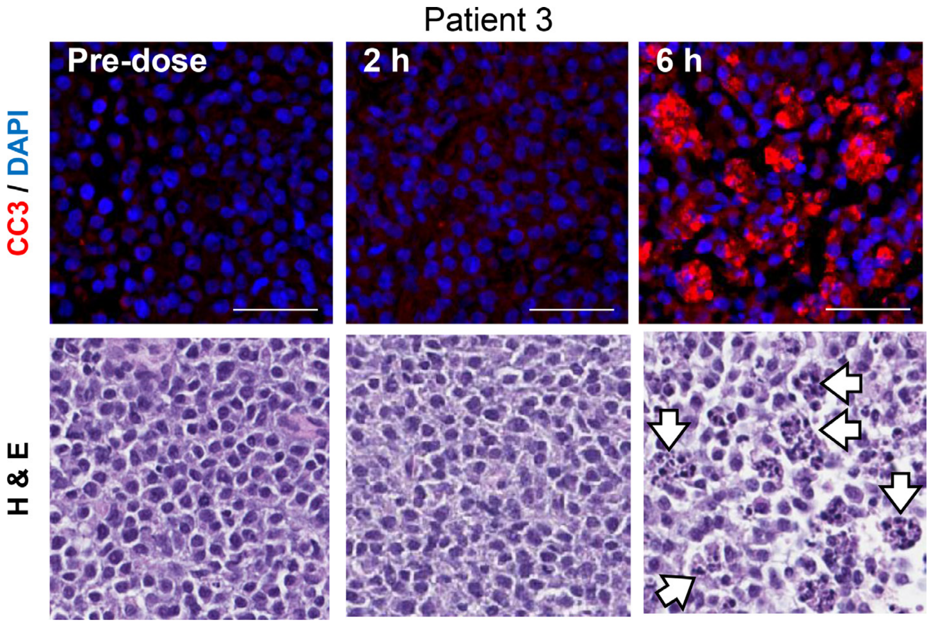

Supplementary Figure 2: Representative CC3 immunofluorescence/DAPI images and H & E–stained images for canine lymphoma patient 3 treated with an investigational agent. White arrows indicate pathologist-annotated "starry-sky" tumor-associated macrophages, which associate with apoptotic tumor cells in some lymphomas. Image quantitation values for specimens from this patient are shown in Figure 2. Scale bars represent 50 μm.
